# Association between OGG1 Ser326Cys and APEX1 Asp148Glu polymorphisms and breast cancer risk: a meta-analysis

**DOI:** 10.1186/1746-1596-9-108

**Published:** 2014-06-03

**Authors:** Qiliu Peng, Yu Lu, Xianjun Lao, Zhiping Chen, Ruolin Li, Jingzhe Sui, Xue Qin, Shan Li

**Affiliations:** 1 Department of Clinical Laboratory, First Affiliated Hospital of Guangxi Medical University, Nanning, Guangxi 530021, China; 2 Department of Occupational Health and Environmental Health, School of Public Health at Guangxi Medical University, Nanning, Guangxi, China; 3 Department of Medicine Research, First Affiliated Hospital of Guangxi Medical University, Nanning, Guangxi, China

**Keywords:** Breast cancer, OGG1, APE1, Polymorphism, Meta-analysis

## Abstract

**Background:**

The base excision repair (BER) pathway removes DNA damage caused by ionizing radiation, reactive oxidative species and methylating agents. OGG1 and APE1 are two important genes in the BER pathway. Many epidemiological studies have evaluated the association between polymorphisms in the two BER genes (OGG1 Ser326Cys and APE1 Asp148Glu) and breast cancer risk. However, the results are inconsistent.

**Methods:**

We searched the electronic databases including PubMed, Embase and Cochrane library for all eligible studies for the period up to February 2014. Data were extracted by two independent authors and pooled odds ratios (ORs) with corresponding 95% confidence intervals (CIs) were used to assess the strength of the association.

**Results:**

A total of 17 studies including 9,040 cases and 10,042 controls were available for OGG1 Ser326Cys polymorphism and 7 studies containing 2,979 cases and 3,111 controls were included for APE1 Asp148Glu polymorphism. With respect to OGG1 Ser326Cys polymorphism, we did not find a significant association with breast cancer risk when all eligible studies were pooled into the meta-analysis. However, in subgroup analyses by ethnicity and menopausal status, statistical significant increased breast cancer risk was found in Asian populations (Cys/Cys vs. Ser/Ser: OR = 1.157, 95% CI 1.013–1.321, *P* = 0.011; Cys/Cys vs. Ser/Cys + Ser/Ser: OR = 1.113, 95% CI 1.009–1.227, *P* = 0.014) and postmenopausal patients (Cys/Cys vs. Ser/Cys + Ser/Ser: OR = 1.162, 95% CI 1.003–1.346, *P* = 0.024). In subgroup analysis according to quality score, source of control, and HWE in controls, no any significant association was detected. With respect to APE1 Asp148Glu polymorphism, no significant association with breast cancer risk was demonstrated in the overall and stratified analyses.

**Conclusions:**

The present meta-analysis suggests that the OGG1 Ser326Cys polymorphism may be a risk factor for breast cancer in Asians and postmenopausal patients. Further large and well-designed studies are needed to confirm this association.

**Virtual Slides:**

The virtual slide(s) for this article can be found here: http://www.diagnosticpathology.diagnomx.eu/vs/1156934297124915

## Background

Breast cancer is currently the most frequently occurring cancer and one of the leading causes of cancer-related death of females worldwide, which has become a major public health challenge [1,2]. Breast cancer is a heterogeneous disease such that they may have different prognoses and respond to therapy differently despite similarities in histological types, grade and stage [3,4]. Because of the heterogeneity of breast cancer, the mechanism of breast carcinogenesis is still not fully understood. It has been well established that exposure to various endogenous and exogenous mutagens or carcinogens played a critical role in the development of breast cancer [5,6]. The exposures can lead to DNA damage which, if remained unrepaired, may result in genetic instability and unregulated cell growth, and eventually breast cancer [7]. The DNA repairing systems, composed of many DNA repair genes, play a critical role in removing damaged genes resulting from endogenous and exogenous mutagenic exposures, and maintaining the genomic integrity and preventing carcinogenesis.

The base excision repair (BER) pathway is one of the most important DNA repair mechanisms responsible for the repair of DNA damage. It is the most common route for removal of small lesions from DNA and is an important part of cellular defense against a large variety of structurally unrelated DNA lesions. It is believed to be the predominant pathway used for removal of oxidized and many alkylated bases [8,9]. BER is initiated by recognition and excision of damaged base by the specific DNA glycosylase. Mammalian cells contain a series of different genes (each with a specialized function), of which 8-oxoguanine glycosylase-1 (OGG1), and apurinic/apyimidinic endonuclease 1 (APE1) genes are two key enzymes in this repair pathway [10]. OGG1 maps on chromosome 3p26.2 and encodes the DNA repair enzyme OGGl responsible for the excision of 8-oxo-7, 8-dihydroguanine (8-oxoG) and other oxidatively damaged DNA bases. The APEX1 gene consists of five exons and four introns spanning 2.21 kb. This gene is located on chromosome 14q11.2–q12. By hydrolyzing 3'-blocking fragments from oxidized DNA, APEX1 produces normal 3'-hydroxyl nucleotide termini that are necessary for DNA repair synthesis and ligation at single- or double-strand breaks [11,12]. Many single nucleotide polymorphisms in the OGG1 and APEX1 gene have been reported, including the commonly occurring Ser326Cys in OGG1 (rs1052133 in dbSNP) and Asp148Glu in APEX1 (rs3136820 in dbSNP). These nonconservative amino acid alterations have been reported to reduce DNA repair activity and consequently increase cancer risk [13,14].

To date, many epidemiological studies have been performed to evaluate the association between OGG1 Ser326Cys and APEX1 Asp148Glu polymorphisms and breast cancer risk, but the results remain conflicting rather than conclusive. With respect to APEX1 Asp148Glu polymorphism, a meta-analysis by Wu et al. [15] found that the APEX1 Asp148Glu polymorphism may not contribute to breast cancer risk, however, they failed to include all eligible studies in the meta-analysis [16,17], which make their conclusions questionable. With respect to OGG1 Ser326Cys polymorphism, two meta-analyses [18,19] investigating the same hypothesis, quite similar in methods and performed almost at the same time, yielded different conclusions. Furthermore, the two previous meta-analyses did not cover all eligible studies [16,17,20-22]. The exact relationship between genetic polymorphisms of OGG1 Ser326Cys and APEX1 Asp148Glu and breast cancer susceptibility has not been entirely established. To provide the most comprehensive assessment of the associations between OGG1 Ser326Cys and APEX1 Asp148Glu polymorphisms and breast cancer risk, we performed an updated meta-analysis of all available studies.

## Methods

### Search strategy

We conducted a comprehensive literature search in PubMed, Embase, and Cochrane library databases for all eligible studies (updated to February 01, 2014) using the following search strategy: (“breast cancer”) and (“OGG1”, “hOGG1”, “APEX1” or “APEX”) and (“polymorphism”, “variation”, “mutation”, “genotype”, or “genetic polymorphism”). There was no restriction on time period, sample size, population, language, or type of report. All eligible studies were retrieved and their references were checked for other relevant studies. The literature retrieval was performed in duplication by two independent reviewers (Qiliu Peng and Shi Yang). When multiple publications reported on the same or overlapping data, we chose the most recent or largest population. When a study reported the results on different subpopulations, we treated it as separate studies in the meta-analysis.

### Selection criteria

Studies were included if they met the following criteria: (1) Case–control studies which evaluated the association between OGG1 Ser326Cys and APEX1 Asp148Glu polymorphisms and breast cancer risk; (2) had an odds ratio (OR) with 95% confidence interval (CI) or other available data for estimating OR (95% CI); and (3) control population did not contain malignant tumor patients. Studies were excluded if one of the following existed: (1) no control population; (2) duplicate of previous publication; and (3) insufficient information for data extraction; (4) Family-based studies of pedigrees with several affected cases per family were also excluded, because their analysis is based on linkage considerations.

### Data extraction

Two investigators (Qiliu Peng and Yu Lu) independently reviewed and extracted data from all eligible studies. To ensure the accuracy of the information extracted, the two investigators checked the data extraction results and reached consensus on all of the items. If different results were generated, they would check the data again and have a discussion to come to an agreement. If these two authors could not reach a consensus, another author (Xue Qin) was consulted to resolve the dispute and a final decision was made by the majority of the votes. Data extracted from eligible studies included the first author, year of publication, country of origin, ethnicity, genotyping method, matching criteria, source of control, breast cancer confirmation, total numbers of cases and controls and genotype frequencies of cases and controls. When data were otherwise unavailable, we contacted the corresponding author by e-mail for original information. Ethnic backgrounds were categorized as Caucasian, Asian, and Africans. When a study did not state the ethnic descendent or if it was impossible to separate participants according to such phenotype, the group reported was termed as “mixed ethnicity”. Menopausal status was divided into premenopausal and postmenopausal and was additionally recorded for the stratified analysis.

### Quality score evaluation

The quality of eligible studies was evaluated independently by two authors (Xue Qin and Qiliu Peng) according to a set of predefined criteria (Additional file 1: Table S1) based on the scale of Thakkinstian et al. [23]. The revised criteria cover the representativeness of cases, source of controls, ascertainment of breast cancer, total sample size, quality control of genotyping methods, and Hardy-Weinberg equilibrium (HWE) in the control population. Disagreements were resolved by consensus. Scores ranged from 0 (lowest) to 10 (highest). Articles with scores equal to or less than 6 were considered “low-quality” studies, whereas those with scores higher than 6 were considered “high-quality” studies.

### Statistical analysis

The strength of the association between OGG1 Ser326Cys and APEX1 Asp148Glu polymorphisms and breast cancer risk was assessed by odds ratios (ORs) with 95% confidence intervals (CIs). The significance of the pooled OR was determined by Z test and a *p* value less than 0.05 was considered significant. The association of OGG1 Ser326Cys and APEX1 Asp148Glu polymorphisms with breast cancer risk was assessed using additive models, recessive model, and dominant model. Heterogeneity assumption was checked by a chi-square-based Q-test [24]. A *P*_*h*_ value equal to or greater than 0.10 for the Q-test indicates a lack of heterogeneity among studies, and so the fixed-effects model was used for the meta-analysis [25]. Otherwise, the random-effects model was used [26]. Subgroup analyses were performed by ethnicity, menopausal status, quality score, source of control, and HWE in controls. Sensitivity analysis was performed by sequential omission of individual studies. For each polymorphism, publication bias was evaluated using a funnel plot and Egger’s regression asymmetry test. The distribution of the genotypes in the control population was tested for HWE using a goodness-of-fit Chi-square test. All analyses were performed using Stata software, version 12.0 (Stata Corp., College Station, TX). All *p* values were two-sided. To ensure the reliability and the accuracy of the results, two authors entered the data into the statistical software programs independently with the same results.

## Results

### Study characteristics

**Figure 1:**
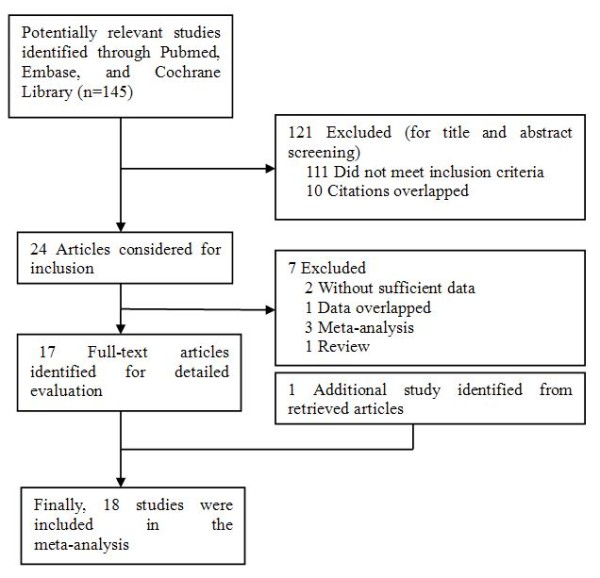
Flowchart of selection of studies for inclusion in meta-analysis.

Based on our search criteria, 24 studies relevant to the role of OGG1 Ser326Cys and APEX1 Asp148Glu polymorphisms on breast cancer susceptibility were identified. Seven of these articles were excluded: one was a review [27], one contained overlapping data [28], two did not present sufficient data for calculating OR and 95% CI [29,30], and three were meta-analysis [15,18,19]. Manual search of references cited in the eligible studies identified 1 additional article [16]. As a result, a total of 18 relevant studies [16,17,20-22,31-43] met the inclusion criteria for the meta-analysis. Among them, two studies [32,42] contained data on two different ethnic groups, and we treated them independently. Therefore, a total of 20 separate comparisons were finally included in the meta-analysis (Figure 1). Of all eligible studies, 13 studies evaluated the OGG1 Ser326Cys polymorphism, 3 studies evaluated the APEX1 Asp148Glu polymorphism, and 4 studies evaluated OGG1 Ser326Cys and APEX1 Asp148Glu polymorphisms simultaneously. Therefore, a total of 17 studies including 9,040 cases and 10,042 controls were available for hOGG1 Ser326Cys polymorphism and 7 studies containing 2,979 cases and 3,111 controls were included for APE1 Asp148Glu polymorphism. Table 1 list all essential information such as the publication year, first author, country, ethnicity, sample size, genotyping methods, source of controls, matching criteria, and breast cancer confirmation for OGG1 Ser326Cys and APEX1 Asp148Glu polymorphisms. The genotype distributions of the controls in 2 studies [32,41] were not consistent with HWE for OGG1 Ser326Cys polymorphism and 1 was not consistent with HWE for APEX1 Asp148Glu polymorphism [17].

**Table 1:** **Characteristics of studies included in this meta**-**analysis**

**First author (Year)**	**Ethnicity**	**Sample size (case/control)**	**Genotyping methods**	**Matching criteria**	**Source of control**	**BC confirmation**	**Quality scores**	**SNP studied**	**HWE(*P *value)**	
									**Ser326Cys**	**Asp148Glu**
Vogel [31]	Caucasian	425/434	Sequencing	Age, menopausal status	PB	NA	7	OGG1 Ser326Cys	0.175	—
Choi [32]	Asian	265/284	PCR-RFLP	NA	HB	Histopatho-	6.5	OGG1 Ser326Cys	0.048	—
Choi [32]	Asian	201/184	PCR-CTPP	Age	HB	Histo-	6	OGG1 Ser326Cys	0.914	—
Huang [16]	Asian	136/232	PCR-RFLP	Age	HB	Patho-	6	OGG1 Ser326Cys APEX1 Asp148Glu	0.525	0.565
Zhang [33]	Caucasian	1571/1244	TaqMan	Age	PB	NA	8	OGG1 Ser326Cys APEX1 Asp148Glu	0.930	0.456
Rossner [34]	Caucasian	1041/1093	FP-TDI	Age	PB	NA	8	OGG1 Ser326Cys	0.857	—
Cai [35]	Asian	1102/1167	TaqMan	Age	PB	NA	8	OGG1 Ser326Cys	0.080	—
Synowiec [36]	Caucasian	41/48	PCR-RFLP	Age	HB	NA	2.5	OGG1 Ser326Cys	0.507	—
Sangrajrang [37]	Asian	506/424	Capillary PCR	Region, menopausal status, drinking, smoking	HB	Histo-	7	OGG1 Ser326Cys APEX1 Asp148Glu	0.627	0.135
Romanowicz-Makowska [38]	Caucasian	100/106	PCR-RFLP	Age	HB	NA	2.5	OGG1 Ser326Cys	0.988	—
Loizidou [39]	Caucasian	1108/1174	TaqMan	Age	PB	Patho-	9.5	OGG1 Ser326Cys	0.499	—
Sterpone [28]	Caucasian	43/34	PCR-SSCP	Age, gender	HB	NA	3	OGG1 Ser326Cys	0.577	—
Ming-Shiean [41]	Asian	401/533	TaqMan	Age	HB	Patho-	6.5	OGG1 Ser326Cys	0.034	—
Roberts [20]	Caucasian	1054/1887	MALDI-TOF	Age, race	PB	Histo-	8	OGG1 Ser326Cys	0.543	—
Smolarz [22]	Caucasian	70/70	PCR-RFLP	Age	HB	Histo-	5	OGG1 Ser326Cys	0.473	—
Kim [17]	Asian	346/351	ASPE	Age, BMI, smoking, drinking	HB	Histo-	6.5	OGG1 Ser326Cys APEX1 Asp148Glu	0.296	0.012
Xie [21]	Asian	630/777	Sequencing	Age, BMI, menopausal status	HB	Patho-	7	OGG1 Ser326Cys	0.161	—
Smith [42]	Caucasian	319/405	TaqMan	Age, race	HB	Histopatho-	7	APEX1 Asp148Glu	—	0.507
Smith [42]	African	53/75	TaqMan	Age, race	HB	Histopatho-	6	APEX1 Asp148Glu	—	0.566
Jelonek [43]	Caucasian	91/412	PCR-RFLP	Age, gender	PB	NA	5	APEX1 Asp148Glu	—	0.092

BC, breast cancer; Histopatho-, Histopathologically confirmed; Histo-, Histologically confirmed; Patho-, Pathologically confirmed; NA, Not available; PB, Population–based; HB, Hospital–based; HWE, Hardy–Weinberg equilibrium in control population; PCR–RFLP, Polymerase chain reaction-restriction fragment length polymorphism; PCR-CTPP, Polymerase chain reaction with confronting two-pair primers; FP-TDI, Template-directed primer extension and detection by fluorescence polarization; PCR-SSCP, Polymerase chain reaction-single strand conformation polymorphism; MALDI-TOF, Matrix-assisted laser desorption/ionization-time of flight mass spectrometry; ASPE, Allele-specific primer extension method.

### Meta-analysis results

**Figure 2:**
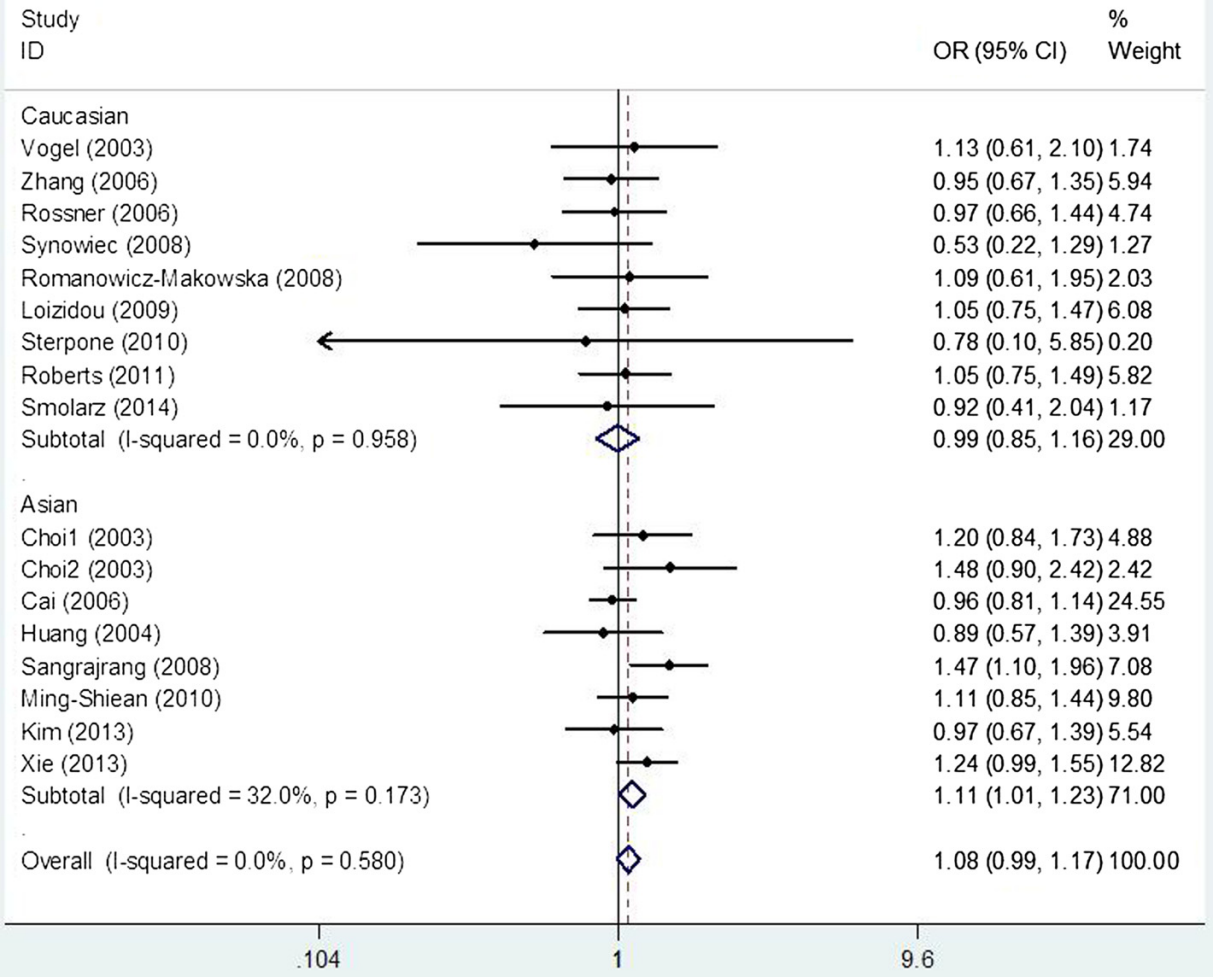
Forest plots of OGG1 Ser326Cys polymorphism and breast cancer risk in subgroup analysis by ethnicity using a fixed-effect model (Recessive model Cys/Cys vs. Ser/Cys + Ser/Ser).

**Figure 3:**
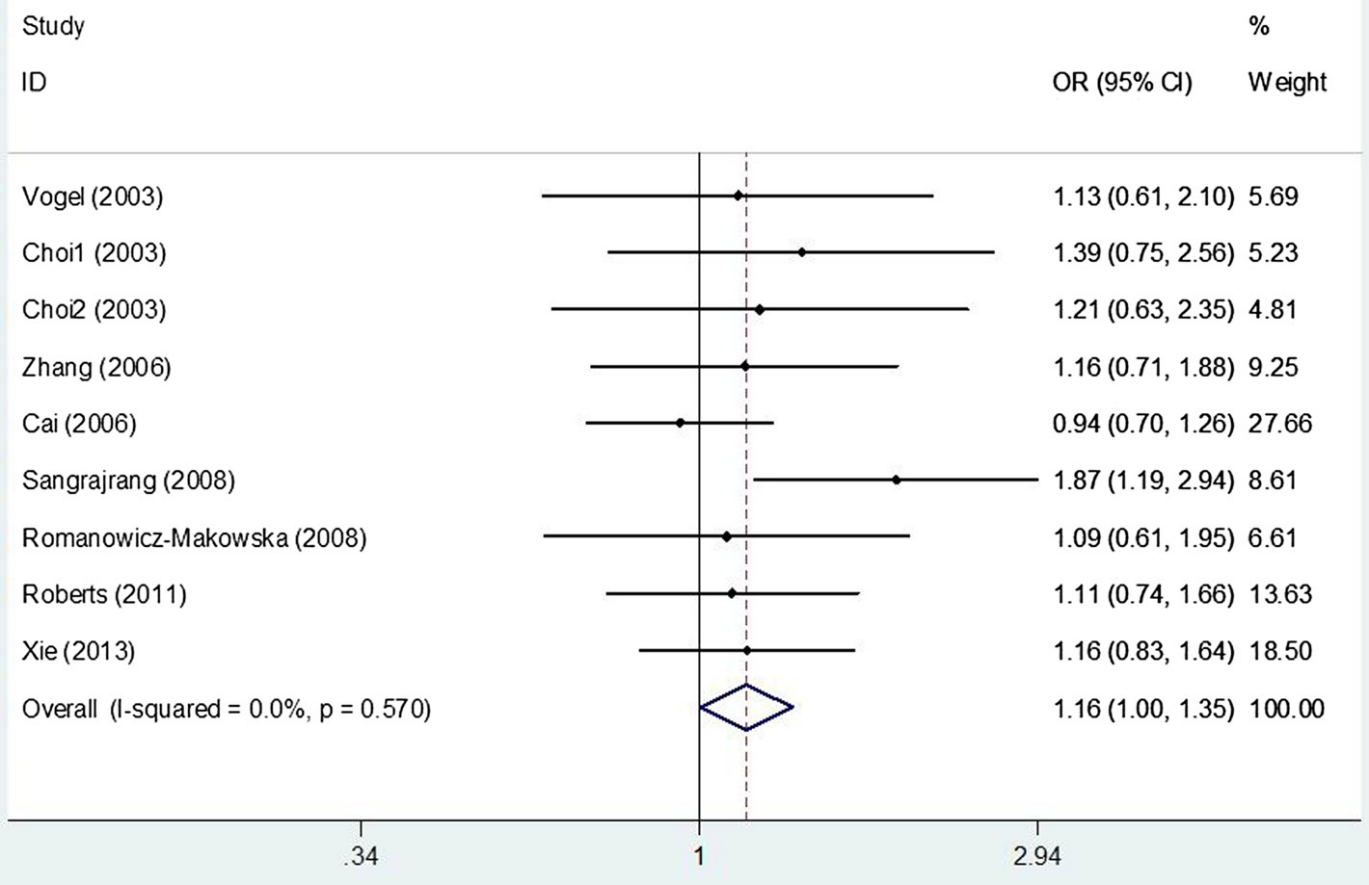
Forest plots of OGG1 Ser326Cys polymorphism and breast cancer risk in postmenopausal patients using a fixed-effect model (Recessive model Cys/Cys vs. Ser/Cys + Ser/Ser).

Table 2 lists the main results of meta-analysis of OGG1 Ser326Cys polymorphism and breast cancer risk. The between-study heterogeneity was not significant in the overall populations and all subgroup analyses (all *P*_*h*_ values were larger than 0.1), thus the fixed-effects model was used to pool the results. Overall, significant elevated breast cancer risk was not found when all studies were pooled into the meta-analysis. When stratified by quality score, source of control, and HWE in controls, significant increased breast cancer risk was also not detected in all subgroups. However, in subgroup analysis by ethnicity, significant increased breast cancer risk was found in Asians (Cys/Cys vs. Ser/Ser: OR = 1.157, 95% CI 1.013–1.321, *P* = 0.011; Cys/Cys vs. Ser/Cys + Ser/Ser: OR = 1.113, 95% CI 1.009–1.227, *P* = 0.014; Figure 2) but not in Caucasians. In stratified analysis by menopausal status, significant increased breast cancer risk was observed in postmenopausal patients (Cys/Cys vs. Ser/Cys + Ser/Ser: OR = 1.162, 95% CI 1.003–1.346, *P* = 0.024; Figure 3) but not in premenopausal subjects.

**Table 2:** **Meta**-**analysis of OGG1 Ser326Cys polymorphism and breast cancer risk**

**Analysis**	**No. of studies**	**Cys/Cys vs. Ser/Ser (Homozygote)**		**Ser/Cys vs. Ser/Ser (Heterozygote)**		**Cys/Cys + Ser/Cys vs. Ser/Ser (Dominant model)**		**Cys/Cys vs. Ser/Cys + Ser/Ser (Recessive model)**	
		**OR (95% CI)**	***P*/*P***_**h**_	**OR (95% CI)**	***P*/*P***_**h**_	**OR (95% CI)**	***P*/*P***_**h**_	**OR (95% CI)**	***P*/*P***_**h**_
Overall	17	1.073(0.968-1.190)	0.180/0.485	0.986(0.923-1.054)	0.679/0.594	1.001(0.940-1.067)	0.971/0.501	1.079(0.993-1.172)	0.074/0.580
Ethnicity									
Caucasian	9	0.956(0.812-1.127)	0.595/0.635	0.964(0.891-1.043)	0.357/0.218	0.967(0.897-1.043)	0.388/0.271	0.995(0.850-1.164)	0.945/0.958
Asian	8	1.157(1.013-1.321)	0.011/0.492	1.043(0.922-1.178)	0.505/0.946	1.085(0.967-1.218)	0.166/0.903	1.113(1.009-1.227)	0.014/0.173
Menopausal status									
Premenopausal	7	1.153(0.957-1.388)	0.134/0.215	1.030(0.897-1.182)	0.677/0.995	1.052(0.923-1.199)	0.449/0.855	1.175(0.929-1.487)	0.178/0.149
Postmenopausal	9	1.154(0.965-1.380)	0.116/0.710	0.962(0.863-1.072)	0.482/0.202	0.994(0.896-1.102)	0.903/0.351	1.162(1.003-1.346)	0.024/0.570
Quality score									
>6	11	1.093(0.980-1.218)	0.110/0.845	0.993(0.928-1.063)	0.841/0.941	1.007(0.944-1.075)	0.829/0.912	1.086(0.995-1.186)	0.065/0.507
≤6	6	0.916(0.664-1.262)	0.590/0.110	0.879(0.667-1.159)	0.361/0.157	0.910(0.702-1.180)	0.478/0.170	1.017(0.789-1.310)	0.899/0.446
Source of control									
HB	11	1.134(0.975-1.320)	0.104/0.164	0.966(0.842-1.107)	0.615/0.384	1.029(0.904-1.170)	0.669/0.238	1.166(0.945-1.307)	0.107/0.462
PB	6	1.023(0.889-1.178)	0.750/0.997	0.992(0.920-1.071)	0.845/0.656	0.993(0.923-1.067)	0.843/0.802	0.987(0.874-1.114)	0.829/0.987
HWE in controls									
Yes	15	1.071(0.960-1.195)	0.218/0.343	0.989(0.924-1.059)	0.757/0.468	1.002(0.939-1.069)	0.961/0.362	1.068(0.976-1.168)	0.465/0.154
No	2	1.086(0.803-1.471)	0.592/0.993	0.929(0.693-1.244)	0.620/0.717	0.993(0.755-1.307)	0.961/0.765	1.142(0.923-1.413)	0.223/0.726

*P*_h_ P values of Q-test for heterogeneity test. OR, odds ratio; CI, confidence intervals; HB, Hospital–based studies; PB, Population-based studies; HWE, Hardy–Weinberg equilibrium.

Table 3 lists the main results of meta-analysis of APEX1 Asp148Glu polymorphism and breast cancer risk. When all 7 studies were pooled into the meta-analysis, there was no evidence of significant association between APEX1 Asp148Glu polymorphism and breast cancer risk (Glu/Glu vs. Asp/Asp: OR = 0.948, 95% CI = 0.817–1.101, *P* = 0.486; Asp/Glu vs. Asp/Asp: OR = 0.975, 95% CI = 0.790–1.204, *P* = 0.814; Glu/Glu + Asp/Glu vs. Asp/Asp: OR = 0.959, 95% CI = 0.803–1.146, *P* = 0.646; Glu/Glu vs. Asp/Glu + Asp/Asp: OR = 0.961, 95% CI = 0.846–1.091, *P* = 0.540). In stratified analyses by ethnicity, menopausal status, quality score, source of control, and HWE in controls, statistically significant association was also not found in all subgroups.

**Table 3:** **Meta**-**analysis of APEX1 Asp148Glu polymorphism and breast cancer risk**

**Analysis**	**No. of studies**	**Glu/Glu vs. Asp/Asp (Homozygote)**		**Asp/Glu vs. Asp/Asp (Heterozygote)**		**Glu/Glu + Asp/Glu vs. Asp/Asp (Dominant model)**		**Glu/Glu vs. Asp/Glu + Asp/Asp (Recessive model)**	
		**OR (95% CI)**	***P*/*P***_**h**_	**OR (95% CI**)	***P/P***_**h**_	**OR (95% CI)**	***P/P***_**h**_	**OR (95% CI**)	***P/P***_**h**_
Overall	7	0.948(0.817-1.101)	0.486/0.508	0.975(0.790-1.204)	0.814/0.028	0.959(0.803-1.146)	0.646/0.074	0.961(0.846-1.091)	0.540/0.572
Ethnicity									
Caucasian	3	1.019(0.850-1.221)	0.842/0.395	0.928(0.670-1.285)	0.651/0.071	0.955(0.717-1.271)	0.751/0.097	1.042(0.897-1.211)	0.590/0.839
Asian	3	0.813(0.620-1.066)	0.135/0.455	1.031(0.681-1.560)	0.886/0.017	0.968(0.680-1.378)	0.855/0.035	0.873(0.604-1.091)	0.142/0.847
African	1	0.948(0.817-1.101)	0.794/—	0.870(0.404-1.870)	0.720/—	0.870(0.426-1.774)	0.701/—	0.933(0.353-2.469)	0.889/—
Menopausal status									
Premenopausal	2	0.895(0.662-1.209)	0.469/0.509	0.938(0.744-1.181)	0.584/0.688	0.921(0.741-1.145)	0.460/0.602	0.927(0.716-1.201)	0.567/0.556
Postmenopausal	2	0.882(0.515-1.510)	0.648/0.096	1.046(0.847-1.290)	0.678/0.644	1.021(0.839-1.243)	0.835/0.323	0.972(0.782-1.208)	0.797/0.113
Quality score									
>6	4	0.946(0.806-1.109)	0.491/0.351	0.999(0.760-1.313)	0.994/0.008	0.973(0.779-1.215)	0.807/0.031	0.952(0.831-1.091)	0.479/0.266
≤6	3	0.968(0.637-1.472)	0.880/0.368	0.898(0.642-1.256)	0.528/0.389	0.907(0.661-1.245)	0.547/0.303	1.024(0.719-1.456)	0.897/0.716
Source of control									
HB	5	0.821(0.659-1.025)	0.081/0.809	0.921(0.669-1.268)	0.612/0.011	0.898(0.697-1.157)	0.405/0.055	0.864(0.709-1.052)	0.146/0.642
PB	2	1.070(0.874-1.309)	0.513/0.398	1.049(0.882-1.248)	0.589/0.538	1.055(0.896-1.242)	0.521/0.450	1.037(0.878-1.226)	0.667/0.562
HWE in controls									
Yes	6	0.941(0.803-1.104)	0.456/0.390	0.930(0.818-1.057)	0.268/0.264	0.929(0.824-1.048)	0.232/0.258	0.989(0.864-1.132)	0.869/0.650
No	1	1.001(0.654-1.531)	0.997/—	1.345(0.904-2.162)	0.211/—	1.362(0.994-1.868)	0.055/—	0.774(0.532-1.126)	0.180/—

*P*_h_ P values of Q-test for heterogeneity test. OR, odds ratio; CI, confidence intervals; HB, Hospital–based studies; PB, Population-based studies; HWE, Hardy–Weinberg equilibrium.

### Sensitivity analysis

Sensitivity analysis was performed by sequential omission of individual studies for both OGG1 Ser326Cys and APEX1 Asp148Glu polymorphisms. For analyses of pooling more than three individual studies, the significance of ORs was not influenced excessively by omitting any single study (data not shown). For the OGG1 Ser326Cys polymorphism, sensitivity analysis was further performed by omitting the studies by Choi et al. [32] and Ming-Shiean et al. [41] in which the control populations were not in accordance with HWE. The significance of all ORs was not altered after excluding these two studies. For the APEX1 Asp148Glu polymorphism, a sensitivity analysis was also further performed by omitting the study by Kim et al. [17] in which the control populations were deviated from HWE, and the significance of all ORs was also not altered.

### Publication bias

**Figure 4:**
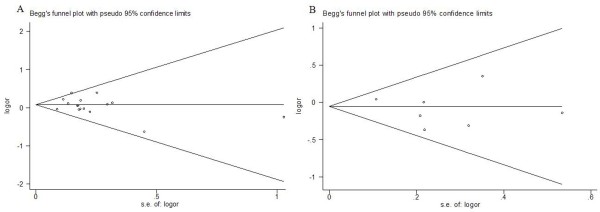
**Funnel plot analysis to detect publication bias.** Each point represents a separate study for the indicated association. **A** Funnel plot for recessive model Cys/Cys vs. Ser/Cys + Ser/Ser of OGG1 Ser326Cys polymorphism in the overall analysis (P = 0.560); **B** Funnel plot for recessive model Glu/Glu vs. Asp/Glu + Asp/Asp of APEX1 Asp148Glu polymorphism in overall analysis (P = 0.504).

Begg’s funnel plot and Egger’s test were performed to access the publication bias in this meta-analysis. The shape of the funnel plot did not reveal any evidence of obvious asymmetry. Then, the Egger’s test was used to provide statistical evidence of funnel plot symmetry. The results still did not suggest any evidence of publication bias in OGG1 Ser326Cys (P = 0.104 for Cys/Cys vs. Ser/Ser; P = 0.187 for Ser/Cys vs. Ser/Ser; P = 0.560 for recessive model Cys/Cys vs. Ser/Cys + Ser/Ser, Figure 4A; and P = 0.339 for dominant model Cys/Cys + Ser/Cys vs. Ser/Ser) and APEX1 Asp148Glu (P = 0.535 for Glu/Glu vs. Asp/Asp; P = 0.789 for Asp/Glu vs. Asp/Asp; P = 0.504 for recessive model Glu/Glu vs. Asp/Glu + Asp/Asp, Figure 4B; and P = 0.766 for dominant model Glu/Glu + Asp/Glu vs. Asp/Asp) polymorphisms.

## Discussion

Maintenance of genomic integrity by DNA repair genes is an essential component of normal cell homeostasis necessary to cell growth, differentiation, and apoptosis [7,44]. Previous evidence indicated that reduced DNA repair capacity, due to various DNA repair gene polymorphisms, is associated with increased risk and susceptibility to cancers [45,46]. BER pathway is one of the most important DNA repair mechanisms responsible for the repair of DNA damage. It was initiated by recognition and excision of damaged base by the specific DNA glycosylase. OGG1 and APEX1 are both central players in the BER pathway. Many epidemiological studies have been performed to evaluate the role of OGG1 Ser326Cys and APEX1 Asp148Glu polymorphisms on breast cancer risk; however, the results remained conflicting and contradictory. The conflicting results are possibly because of the small effect of OGG1 Ser326Cys and APEX1 Asp148Glu polymorphisms on cancer risk or the relatively low statistical power of individual published studies. To provide the most comprehensive assessment of the association between OGG1 Ser326Cys and APEX1 Asp148Glu polymorphisms and breast cancer risk, we performed this meta-analysis of all available studies. Our results suggested that the OGG1 Ser326Cys polymorphism was significantly associated with increased breast cancer risk in Asian populations and postmenopausal patients. However, our data did not support a significant association between APEX1 Asp148Glu polymorphism and breast cancer risk both in the pooled analysis and stratified analyses.

This finding may be biologically plausible. Oxidative DNA damage induced by reactive oxygen species (ROS) is involved in the process of carcinogenesis. Reactive oxygen species could be generated from estrogen metabolism through catechol estrogen redox cycling [47,48]. If not quenched, these reactive oxygen species may cause oxidative DNA damage and increase breast cancer risk. It has been suggested that 8-hydroxyguanine, a major product of oxidative DNA damage, plays an important role in carcinogenesis given its abundant and highly mutagenic properties [49]. 8-Hydroxyguanine is subjected to BER, especially via the 8-oxoguanine DNA glycosylase (OGG1) catalyzing the release of 8-hydroxy-2'-deoxyguanosine and the cleavage of DNA at the AP site. Variants in the OGG1 gene might alter protein structure or function or create alternatively spliced proteins which may influence BER efficiency and hence affect individual susceptibility to cancers. It was reported that the OGG1 326Cys variant enzyme have lower activity than the 326Cys enzyme [50]. More importantly, the homozygous variant genotype OGG1 326Cys has been shown associated with increased risk for many different types of cancers, including colorectal cancer [51], hepatocellular carcinoma [52] and lung cancer [53].

In the stratification analysis of ethnicity, we found an evidence for the association between the hOGG1 Ser326Cys polymorphism and breast cancer susceptibility among Asians but not Caucasians. A possible reason for the differences might be the different genetic backgrounds and gene–environment interactions. We observed a wide variation of the Cys allele frequencies of control resources in Asian (0.52) and Caucasian (0.24) population that were very close to that obtained from the HapMap Project (0.50 for CHB and 0.22 for CEU), and this different allele frequency might account for the discrepancies between the hOGG1 Ser326Cys polymorphism and breast cancer susceptibility among different ethnicity.

Previous evidence suggested that the menopausal status was one of the important risk factors for the development of breast cancer. So we carried out subgroup analysis according to menopausal status in this meta-analysis. Our results suggested a significant increased breast cancer risk in postmenopausal patients (Cys/Cys vs. Ser/Cys + Ser/Ser: OR = 1.162, 95% CI 1.003–1.346, *P* = 0.024) but not in premenopausal subjects. Though results from available studies investigating OGG1 Ser326Cys polymorphism and breast cancer risk have been inconsistent, our results are consistent with the results of the large sample study by Sangrajrang et al. [37]. The most possible reason for this discrepancy in premenopausal and postmenopausal patients is that the OGG1 Ser326Cys polymorphism plays different roles in repairing oxidative DNA damage resulting from estrogen metabolism in patients with different menopausal status.

Some limitations of this meta-analysis should be addressed. First, in subgroup analysis by ethnicity, the included studies regarded only Asians and Caucasians for OGG1 Ser326Cys polymorphism. Data concerning other ethnicities such as Africans were not found. Thus, additional studies are warranted to evaluate the effect of this functional polymorphism on breast cancer risk in different ethnicities, especially in Africans. Second, our results were based on unadjusted estimates. We did not perform the analysis adjusted for other covariates such as age, obesity, drinking and smoking status, use of contraceptives, environment factors, and so on, because of the unavailable original data of the eligible studies.

## Conclusions

The meta-analysis provided a more precise estimation based on larger sample size compared with the individual studies and previous meta-analysis. Our study suggested that OGG1 Ser326Cys polymorphism might contribute to breast cancer risk, especially in Asian populations and postmenopausal patients. In order to further verify our findings, large well designed epidemiological studies are warranted.

## Abbreviations

HWE: Hardy–Weinberg equilibrium; BER: Base excision repair; OGG1: Human 8-oxogunaine glycosylase-1; APEX1: Apurinic/apyrimidinic-endonuclease-1; SNP: Single nucleotide polymorphism; OR: Odds ratio; CI: Confidence interval.

## Competing interest

The authors declare that they have no competing interest.

## Authors’ contributions

QP, XQ, YL, XL performed the literature search, data extraction, statistical analysis and drafted the manuscript. QP, XL, JS, and RL participated in data extraction. QP, SL, XQ, ZC supervised the literature search, data extraction, statistical analysis and drafted the manuscript. All authors read and approved the final manuscript.

## Supplementary Material

Additional file 1: Table S1 Scale for Quality Assessment. Click here for file
