# Supplementary material for: Association between OGG1 Ser326Cys and APEX1 Asp148Glu polymorphisms and breast cancer risk: a meta-analysis
Source: Diagn Pathol. 2014 Jun 3;9:108. doi: 10.1186/1746-1596-9-108 (PMC4064811; doi:10.1186/1746-1596-9-108)
Supplement: Additional file 1: Table S1 — Scale for Quality Assessment. [file 1746-1596-9-108-S1.doc]

Table S1. Scale for Quality Assessment

| Criteria | Score |
| --- | --- |
| Representativeness of cases |  |
| Selected from cancer registry or multiple cancer center sites | 2 |
| Selected from oncology department or cancer institute | 1 |
| Selected without clearly defined sampling frame or with extensive inclusion/exclusion criteria | 0 |
| Source of controls |  |
| Population or community based | 2 |
| Both population-based and hospital-based/healthy volunteers/blood donors | 1.5 |
| Hospital-based controls without breast cancer | 1 |
| Cancer-free controls without total description | 0.5 |
| Not described | 0 |
| Ascertainment of breast cancer |  |
| Histological or pathological confirmation | 2 |
| Diagnosis of breast cancer by patient medical record | 1 |
| Not described | 0 |
| Sample size |  |
| >1000 | 2 |
| 200-1000 | 1 |
| <200 | 0 |
| Quality control of genotyping methods |  |
| Clearly described a different genotyping assay to conﬁrm the data | 1 |
| Not described | 0 |
| Hardy-Weinberg equilibrium |  |
| Hardy-Weinberg equilibrium in controls | 1 |
| Hardy-Weinberg disequilibrium in controls | 0.5 |
| No checking for Hardy-Weinberg disequilibrium | 0 |
